# Developing Traditional Chinese Medicine in the Era of Evidence-Based Medicine: Current Evidences and Challenges

**DOI:** 10.1155/2015/425037

**Published:** 2015-04-08

**Authors:** Foon Yin Fung, Yeh Ching Linn

**Affiliations:** ^1^Traditional Medicine Information Service, Department of Pharmacy, Singapore General Hospital, Singapore; ^2^Department of Haematology, Singapore General Hospital, Academia, 20 College Road, Singapore 169856

## Abstract

Evidence-based medicine (EBM), by integrating individual clinical expertise with the best available clinical evidence from systematic research, has in recent years been established as the standard of modern medical practice for greater treatment efficacy and safety. Traditional Chinese Medicine (TCM), on the other hand, evolved as a system of medical practice from ancient China more than 2000 years ago based on empirical knowledge as well as theories and concepts which are yet to be mapped by scientific equivalents. Despite the expanding TCM usage and the recognition of its therapeutic benefits worldwide, the lack of robust evidence from the EBM perspective is hindering acceptance of TCM by the Western medicine community and its integration into mainstream healthcare. For TCM to become an integral component of the healthcare system so that its benefits can be rationally harnessed in the best interests of patients, it is essential for TCM to demonstrate its efficacy and safety by high-level evidence in accordance with EBM, though much debate remains on the validity and feasibility of applying the EBM model on this traditional practice. This review aims to discuss the current status of research in TCM, explore the evidences available on its efficacy and safety, and highlight the issues and challenges faced in applying EBM to TCM.

## 1. Introduction

Evidence-based medicine (EBM) is defined as “the conscientious, explicit, and judicious use of current best evidence in making decisions about the care of individual patients.” The practice of evidence-based medicine means integrating individual clinical expertise with the best available external clinical evidence from systematic research [1]. In just over two decades since its first introduction, the EBM movement has created a paradigm shift to become the standard of modern medical practice, elevating it to a higher level and with greater accuracy, efficacy, and safety.

Traditional Chinese Medicine (TCM), on the other hand, evolved as a system of medical practice from ancient China more than 2000 years ago. In contrast to the disease-targeted approach adopted by Western medicine where there is a standard treatment protocol defined for each disease entity, TCM takes a holistic approach in treating the individual with customized treatment based on the concept of “Syndrome Differentiation.” The basic theories of TCM were derived from the Chinese philosophy of Yin-Yang and Five Elements, and its fundamental concepts such as the Zang-fu (viscera) concept, *Qi* (vital energy), and meridians have yet to be mapped by scientific equivalents or clearly elucidated in scientific terms.

While TCM is widely practiced in Asian populations such as China, Hong Kong, Taiwan, and Singapore, many non-Asian countries have, in recent decades, also recognized the huge therapeutic potential of this traditional practice and have been actively tapping into the benefits of TCM so as to provide patients with an additional option in their health management. In 1991, a TCM hospital was opened in KoÈtzting, Germany, whereby trained TCM physicians from China administered treatment according to the traditional practice [2]. In the United States, UCLA Center for East-West Medicine was founded in 1993 to provide integrative medicine treatment [3]. In 1998, the National Institutes of Health within the US Department of Health and Human Services set up the National Center for Complementary and Alternative Medicine with the mission to “define, through rigorous scientific investigation, the usefulness and safety of complementary and alternative medicine interventions (including TCM) and their roles in improving health and health care” [4]. In 2007, the National Institute of Complementary Medicine at the University of Western Sydney was established by the Australian and New South Wales Governments with a research focus on Chinese medicine via laboratory analysis, clinical trials, and translation into practice [5]. In 2014, Cleveland Clinic opened a hospital-based Chinese herbal therapy clinic in their Center for Integrative Medicine to provide supplementary options for patients seeking a holistic, natural approach to their care [6]. According to interim data from 2nd WHO TRM global survey as of 11 June 2012, 80% of the 129 member states now accept the use of acupuncture as a treatment modality [7].

Despite the growing prevalence of TCM usage and the worldwide interest in its therapeutic benefits, the fundamental issue hindering its acceptance by the Western medicine community and integration into mainstream healthcare is still the lack of robust evidence from the EBM perspectives. In order for TCM to be rationally used for patients who may benefit from it, it is essential for TCM to demonstrate its efficacy and safety by high-level evidence using methods which are accepted in the evaluation of mainstream medicine, that is, in accordance with evidence-based medicine. In the EBM system, different types of evidences are prioritized into a set of hierarchical levels. Randomized controlled trials (RCTs) or systematic reviews of RCTs are the golden standard for the highest level of evidence, followed by other types of evidences such as cohort studies, case-control studies, case series, case reports, animal/*in vitro* studies, and expert opinion. Whether the EBM model can be applied to TCM has long been a subject of debate and an area where much effort has been focused on.

This review aims at (1) discussing the current status of research in TCM, (2) exploring the evidences available on the efficacy and safety of Chinese herbal medicine and acupuncture, and (3) highlighting the issues and challenges faced in applying EBM to TCM.

## 2. Current Status of Research on TCM Intervention

Vast amount of basic research has been conducted on TCM herbs, mostly in characterizing the multiple-herb constituents, isolating active components from the herbs, and testing their pharmacological activities in the pursuit of new drug discovery. The US Pharmacopeial Convention published the Herbal Medicines Compendium, which is an online database that provides such standards for herbs. Each herbal monograph contains specifications including tests for critical quality attributes of the herbal ingredient, as well as analytical test procedures and acceptance criteria for specified tests [8]. In China, the TCM systems of pharmacology database and analysis platform were developed as a comprehensive source of herb pharmacochemistry, pharmacokinetic properties, and so forth to help drive drug discovery from herbal sources [9]. Two famous examples of active ingredients effectively extracted from TCM and since proven to be clinically successful are artesunate derived from* Artemisia annua* used for severe malaria [10, 11] and arsenic trioxide used for treatment of acute promyelocytic leukaemia [12, 13]. Taking up the challenge of fulfilling the stringent requirements of good clinical practice, a few TCM formulations are currently undergoing FDA clinical trials. These include Danshen dripping pill (*Salvia miltiorrhiza*,* Radix notoginseng*, and Borneol) for stable angina in phase III trial, Kanglaite injection (coix seed oil and excipients) for cancer, Xuezhikang capsule (red yeast) for hyperlipidemia, and Fuzheng Huayu capsule (*Salvia miltiorrhiza*, peach seed, pine pollen,* Gynostemma pentaphylla*, and* Schisandra chinensis*) for liver fibrosis in phase II trials [14].

Quantitatively, there is no shortage of clinical studies in TCM conducted worldwide. A search on the Clinicaltrials.gov found 436 trials on TCM and 607 acupuncture trials globally (as of November 2014), while a search on online scientific journal portals, for example, Pubmed, Chinese National Knowledge Infrastructure (CNKI), yielded more than ten thousand research papers covering a wide spectrum of research topics studying TCM from various approaches. The critical problem, however, lies in the dearth of good quality evidence and lack of strong research rigor in the plethora of studies published, which undermine the credibility of the evidences.

A screening of all the TCM-related systematic reviews published by the Cochrane database to date found that almost all of the reviews commented on the lack of high quality clinical trials done with good methodology, thereby negating any definitive conclusion on the efficacy of herbal or acupuncture interventions. Some of the specific criticisms cited in these reviews included lack of trials that tested the same herbal medicine, lack of details on cointerventions, unclear methods of randomisation, poor reporting, high risks of bias, and the need for well-designed and conducted trials with sufficient numbers of participants to perform subgroup analyses [15]. The lament on the suboptimal quality of clinical studies on TCM was also echoed by two systematic reviews of reports on RCTs of TCM published in China from 1999 to 2004 and those indexed in the CNKI database from 2005 to 2012, respectively. The former found that the mean Jadad score (a scoring system for the methodology quality of a clinical trial on a scale of 0 to 5) of the 7422 RCTs identified was 1.03 overall, with a mean score of only 0.85 in 1999 (746 RCTs) and 1.20 in 2004 (1634 RCTs) [16]. The latter concluded that although the quality of reporting RCTs of TCM has improved in 2011-2012 (2861 trials) compared to 2005–2009 (4133 trials), the percentages of high quality reports remained low-mean Jadad scores of 1.22 versus 1.25, with only 15.15% and 19.71% of the reports having a score of 2 [17]. These results highlighted the present state of huge disparity between the quantity and quality of TCM RCTs and demonstrated that much more work needs to be done to improve on the latter aspect.

## 3. Some Challenges of Applying EBM to TCM Clinical Trials

One fundamental challenge in conducting valid RCTs in TCM is the batch-to-batch variation of the active constituents contained in the herbal formulation used. Product-to-product variation arising from different manufacturers, brands, or formulation further hinders the fair comparison of the findings among similar studies. Such inconsistencies may result in lack of reproducibility between clinical trials and even challenge the validity of generalizing the findings of clinical trials to routine use. However this is something that can potentially be overcome by technological advancement. Newly developed analytical tools and techniques have made it possible to profile the constituents of herbs, for example, by using high-pressure liquid chromatography to establish a chemical fingerprint of the herb, where the profiles of different batches can be compared to ensure no significant variability [18]. Precise reporting of such information by clinical trials has to be implemented to assure validity of TCM trials. In parallel, legislation to define the required standard of proprietary products will eventually be required for acceptance of the clinical trial findings and efficacy of TCM products by the Western medicine practitioners.

Another challenge is the difficulty in creating an appropriate placebo for multiple-herb herbal decoctions, which compromises the effectiveness of blinding. While it is feasible to create placebos for capsules, pills, and tablets, it is technically challenging to create an indistinguishable placebo for a multiple-herb formulation in the form of decoction. However, with multiple-herb formulations in the form of granules being more favored over decoction due to its convenience of use, it is not impossible to overcome the aforementioned challenge. In addressing this need for good quality placebo in TCM clinical research, one recent research in China has described a novel technique which claims feasibility in creating a good quality placebo in granule form that has similar appearance, smell, and taste as the original preparation but with no pharmacological activity [19]. Besides herbal medicine, designing appropriate placebo for acupuncture trials is yet another challenge, though researchers have come up with various control measures such as nonacupuncture inert controls, placebo acupuncture, sham acupuncture, real acupuncture with a decoy treatment, waiting list controls, standard care controls, and adjunctive care comparisons [20].

Incorporating TCM principles in an RCT poses another challenge as it appears that the standardized treatment approach required by RCTs is in discordance with the individualized treatment approach inherent in TCM practice. This difference in approach, however, is not entirely irreconcilable. In order to accommodate TCM principle, that is, replicating the diagnosis using “Syndrome Differentiation” and giving the corresponding individualized treatment within the rigorous framework of EBM, a systematic process for assessing symptoms and signs, the identification, and the quantification of TCM syndrome for a sample group of study subjects with the same Western medical diagnosis can be applied before and after treatment [21]. To quantify a syndrome for diagnosis, the signs and symptoms of each subject could be tabulated and matched against the typical signs and symptoms which characterize a particular TCM syndrome. The percentage match between the two could then serve as the score for a certain syndrome and a corresponding treatment protocol would be administered, allowing an “individualized” treatment in a standardized and reproducible way [21]. One way of assessing the intervention effects could be done by comparing the change in scores.

## 4. Efforts to Improve the Quality of TCM Research

It is encouraging that the need to modernize the methodology of clinical trials in TCM is being recognized and actively addressed. A group of Chinese medicine researchers, practitioners, journal editors, and epidemiologists have established the Chinese CONSORT Group for TCM and proposed the Consolidated Standards for Reporting Trials (CONSORT) of Traditional Chinese Medicine [22]. To improve the quality of TCM research, some of the recommendations made by the group include the precise reporting of TCM interventions and outcome measures detailed as follows [22, 23].

*Precise Reporting of TCM Interventions in RCTs*

- Single Chinese herbal medicine-based/formula-based/extraction-based intervention:
  1. name, dosage format, and registration;
  2. composition and quality of intervention;
  3. pharmaceutical processing and quality control;
  4. stability of final product and quality control;
  5. function and safety description;
  6. dosage and treatment course;
  7. control group.
- Active compound-based TCM drug intervention:
  1. name of active compound(s);
  2. original source of active compounds(s);
  3. the brief process obtaining active compound(s);
  4. percentage of active compound in final product;
  5. added materials and their quality and quantity control.

*Precise Reporting of Outcome Measures of RCTs of TCM*

1. Identifying the primary and secondary outcomes based on the purpose and hypothesis of the trial;
2. defining the primary and secondary outcomes clearly;
3. presenting the rationale of selection;
4. presenting the method with aims to standardize the assessment process;
5. presenting the method to improve the reliability of assessment;
6. stating the termination criteria in the trial.

More recently in 2009, the Good Practice in Traditional Chinese Medicine Research Association was launched. This was the European Union's first coordination action dedicated to TCM research, funded by the European Commission under its 7th Framework Programme to coordinate EU-China dialogues and collaborations in TCM research. This project has since engaged more than 200 scientists and clinicians in discussions on good practice issues related to various aspects of Chinese herbal medicine and acupuncture research [24].

## 5. Examples of Level 1 Evidences Supporting TCM Intervention

Despite the paucity of high quality evidences available, there are nevertheless a few examples of well-conducted RCTs with precise reporting, which attested to the feasibility of producing high quality evidence for TCM and provided valuable insight into this challenging undertaking.

### 5.1. Efficacy of TCM Formulation in Irritable Bowel Syndrome, 1998

In a study on patients with irritable bowel syndrome (IBS) [25], patients were randomly allocated to 1 of 3 treatment groups: individualized Chinese herbal formulations (*n* = 38), a standard 20-herb Chinese herbal formulation (*n* = 43), or placebo (*n* = 35), all in capsule form. Patients were treated for 16 weeks and evaluated regularly by a traditional Chinese herbalist and a gastroenterologist. In this randomized, double-blinded, placebo-controlled trial, TCM formulation was shown to be effective in the management of IBS. Patients who received the standard formulation fared best during the course of treatment, while patients who received the individualized formulations maintained their improvement up to 14 weeks beyond the treatment period. This study is a good illustration of the potential efficacy of TCM for common conditions such as IBS which to date has no good solution by Western medicine other than symptomatic treatment. One unique feature of this study is that it compared the effect of standardized TCM formulation with individualized formulation. TCM practice has always emphasized on individualized treatment and syndrome-directed modification of TCM formulation regularly throughout the course of treatment, which may not be cost-effective or convenient from the patients' perspective. Further trials incorporating such comparisons would help to shed light on the relative superiority of customized treatment over standard formulation.

### 5.2. Efficacy of Maxingshigan-Yinqiaosan as Compared to Oseltamivir in Treating H1N1 Influenza, 2011

A prospective, nonblinded, randomized, controlled, multicenter trial was conducted in young patients in eleven hospitals from 4 provinces in China to compare the efficacy and safety of Oseltamivir and a TCM decoction “Maxingshigan-Yinqiaosan” in treating uncomplicated H1N1 influenza [26]. “Maxingshigan-Yinqiaosan” is a 12-herb formulation combining two classical TCM formulae for their “diaphoretic and heat-clearing” activities to treat respiratory tract infection. Subjects were randomized into four groups: Oseltamivir group (*n* = 102), Maxingshigan-Yinqiaosan group (*n* = 103), Oseltamivir plus Maxingshigan-Yinqiaosan group (*n* = 102), and control group with no intervention (*n* = 103). The study found that Oseltamivir and “Maxingshigan-Yinqiaosan,” whether used alone or in combination over a course of 5 days, resulted in a comparable time to fever resolution in patients with H1N1 influenza virus infection, superior to control group. This data therefore suggests that “Maxingshigan-Yinqiaosan” may be used as an alternative treatment to Oseltamivir for H1N1 influenza virus infection. This trial also illustrates two important facts: that the TCM formulation is as effective as Oseltamivir and that it is safe to be used in combination with Oseltamivir. The former is of relevance in terms of cost consideration, and the latter is relevant in terms of safety for the common layman practice of consuming both Western and traditional medicines with the hope of attaining enhanced therapeutic effect.

### 5.3. CHInese Medicine Neuroaid Efficacy on Stroke Recovery (CHIMES) Study, 2013

MLC601 (Trade name: Neuroaid) is a TCM formulation “Danqipiantan jiaonang” containing 9 herbs and 5 animal components [27]. A systematic review of six RCTs provided evidence that MLC601 could be effective in improving functional independence and motor recovery as an add-on to standard treatment and is safe for patients with primarily nonacute stable stroke [28]. The CHInese Medicine Neuroaid Efficacy on Stroke recovery (CHIMES) study, a multicentre, randomized, double-blinded, placebo-controlled trial (*n* = 1099) comparing MLC601 with placebo in patients with acute ischemic stroke of intermediate severity in the preceding 72 hours, is one of the largest-scale TCM trials in the recent years with subject recruitment from various countries in South East Asia [29]. Although MLC601 is statistically no better than placebo in improving outcomes at 3 months when used among patients with acute ischemic stroke of intermediate severity, a post hoc analysis found that the MLC601 group had significantly less early vascular events compared to the placebo group [30]. The safety profile of MLC601 was also validated by the lack of significant difference in adverse events between treatment and placebo group. This study illustrated a few important lessons: while dispelling unrealistic hope on expedited recovery, it revealed the unexpected findings of reduced vascular events of a magnitude much larger than what aspirin has been shown to produce [31]. It also exemplified a well-conducted cum precisely reported TCM trial done in compliance with good clinical practices. Results from the ongoing extension study [32] investigating the longer-term efficacy over a 2-year follow-up from the initial course of intervention are eagerly awaited. A positive finding will be indicative of the value in conducting long-term assessments after initial short-course therapy, a method which may be relevant in evaluating TCM efficacy since TCM interventions aim at restoring balance and may thus have the potential to achieve long-term and sustained benefit rather than temporary symptomatic relief.

### 5.4. Efficacy of Tianqi Capsule in Patients with Impaired Glucose Tolerance, 2014

In a double-blind, randomized, placebo-controlled, multicenter trial, Tianqi capsule containing 10 TCM herbs was found to significantly decrease the incidence of progression to type 2 diabetes mellitus in subjects with impaired glucose tolerance, after a course of 12-month treatment (*n* = 210) as compared with the placebo group (*n* = 210) [33]. This particular study is laudable in that it reported in detail the subject inclusion and exclusion criteria and the randomization and blinding method as well as adverse events, which are often omitted or not elaborated on in most TCM clinical studies. It also conducted and described the chemical analysis of the composition of Tianqi capsule, quantifying the constituents of the formulation, which is in line with the precise reporting recommendations to improve the quality of TCM studies [22].

### 5.5. Efficacy of Acupuncture for Pain Management Based on Systematic Reviews

A Cochrane review of 33 trials (*n* = 2293) found consistent evidence that acupuncture provides additional benefit in the treatment of acute migraine attacks compared to routine care and that acupuncture is at least as effective as or possibly more effective than prophylactic drug treatment and has fewer adverse effects. The review recommended acupuncture as a possible treatment option for patients who are willing to undergo this treatment [34]. Another review of 11 trials (*n* = 2317) also concluded that acupuncture could be a valuable nonpharmacological tool in patients with frequent episodic or chronic tension-type headaches [35]. A review on efficacy of acupuncture on neck pain concluded that there is also moderate evidence that acupuncture is better at relieving neck pain compared with some sham treatments. It found that at short-term follow-up, patients who received acupuncture reported less pain than those on a waiting list and that acupuncture is more effective than inactive treatments for relieving pain after treatment and this effect is maintained at short-term follow-up [36]. A systematic review (12 trials, *n* = 1763) reported that acupuncture use is associated with significant reductions in pain intensity and improvement in functional mobility as well as quality of life in patients with osteoarthritis [37].

While these level 1 evidences on the whole prove the efficacy of certain TCM treatments, one shortcoming among these studies is the lack of incorporation of TCM principles in the study design. For example, with the exception of the IBS study, the principal concept of “Syndrome Differentiation” which guides TCM diagnosis and treatment was not applied. Subjects were recruited based on their diagnosis by Western medicine without further subcategorization into different TCM syndromes for which different formulations would be required correspondingly. Under such circumstances, the assessment of the efficacy of TCM treatment may be flawed because the methodology does not replicate the real-life practice of TCM, and any lack of efficacy demonstrated could arguably be a result of the deviation from the well-tested TCM principles. As advocated by some, study designs guided by TCM theory are necessary to validate and improve future RCTs in TCM [38].

## 6. Current Status of Available Evidence on Safety Issues of TCM Usage

Efficacy issues aside, one much neglected aspect of TCM is the safety issues of its usage, for which there is currently little available evidence but warrants great attention. We believe that the prerequisite for TCM to be accepted into mainstream healthcare lies in addressing the safety concerns including adverse herb reaction and herb-drug interactions. The following sections therefore elaborate on evidence available on the safety aspects of TCM.

As highlighted by the Chinese CONSORT for TCM, it is important for RCTs to transparently report not only efficacy but also all the related adverse effects [39]. In general, adverse effects (AE) of the TCM interventions can be classified into 5 types as summarized below [39]. We will discuss more toxicity inherent to the herbs and the important issue of herb-drug interaction.

*Types of Adverse Effects of TCM Interventions*

1. AEs under proper TCM principles and guidelines, for example, acute/chronic toxicity and allergy;
2. AEs due to improper usage without following TCM principles, involving without following the TCM therapeutic principles, over-dosage, improper processing and preparation methods, and improper formula strategy;
3. AEs due to contamination, such as heavy metal and pesticides contaminations in TCM interventions and intentional or unintentional contamination with drugs;
4. AEs due to replacement of TCM herbs;
5. AEs due to drug-herb interaction.

### 6.1. Toxicity

Evidence of adverse effects can come from various levels, from careful monitoring in RCTs, to case series and case reports on observed toxicity. Evidence from animal or* in vitro* studies may be deemed as low level from the perspective of proving efficacy, but their role in providing safety information is much greater. Biomedical research on herb pharmacology and toxicity are crucial to ensure the safe and appropriate use of herbs. Conclusive evidences from modern studies should be continually updated into the Chinese herbal pharmacopoeia and taken into consideration in the current practice of TCM regardless of whether they were mentioned in ancient Chinese literature.

“Chinese Herb Nephropathy” is the unfortunate term coined after the misuse of Guang Fang Ji (*Aristolochia fangchi*) instead of Han Fang Ji (*Stephania tetrandra*) in slimming pills in a few European centers, which led to interstitial nephritis, renal failure, and urothelial carcinoma [40]. This is an example of evidence of toxicity learned the hard way. While ancient TCM literature did not caution against such toxicity, with modern technology we now know that Aristolochic acid is one of the most potent carcinogens known [41]. Such evidence of toxicity based on laboratory research is most clinically relevant and the physician's knowledge of such should prevent catastrophic adverse effects in clinical practice.

Another example of toxic alkaloids found in TCM herbs which are still widely used clinically is aconitine which is found in* Aconitum* species such as Fuzi/Chuanwu (*Aconitum carmichaelii*) and Caowu (*Aconitum kusnezoffii*). These herbs are often used for their analgesic and cardiotonic properties. Aconitine poisoning resulting from overdosing or inappropriate processing of herbs before consumption could lead to cardiovascular, gastrointestinal, and neurological toxicities such as palpitations, arrhythmias, hypotension, and perioral and limb paraesthesia [42, 43]. Another known toxic herbal compound is ephedrine, the main active ingredient in Mahuang (*Ephedra sinica*) which is frequently used in respiratory condition for its antiasthmatic activity. Studies have found that misusing Mahuang at higher dosages could lead to hypertension and cardiac arrhythmia due to its sympathomimetic activity [44]. These are examples where the known pharmacological and toxicological properties of herbs, corroborated by even small case series which are low in the hierarchy in the pyramid of evidence levels, can constitute important evidence for developing guidelines on the safe dosage and contraindications in the use of herbs.

Apart from the above examples which are known to have toxicities, certain TCM herbs which are not known to be toxic may also pose health risks if consumed inappropriately. For example, Gancao (*Glycyrrhiza uralensis*) is a frequently used herb in TCM formulations for its *Qi*-tonifying effect. However, evidence provided from biochemical studies demonstrated that Gancao has corticosteroidal activity. Glycyrrhizin, the principal active ingredient in Gancao, and its hydrolysed metabolite Glycyrrhetinic acid are inhibitors of the oxidative function of the 11*β*-hydroxysteroid dehydrogenase, thereby preventing the metabolism of cortisol. As such, cortisol could then bind to mineralocorticoid receptors, leading to pseudoaldosteronism, resulting in hypertension and hypokalemia [45]. The clinical significance of this experimental finding was ascertained by a few adverse case reports of pseudoaldosteronism caused by consumption of Gancao at high doses [46]. Further work to better define the safe dose of Gancao in prolonged usage will provide important evidence for physicians to utilize this herb safely in modern day practice.

The use of Huang Lian (*Rhizoma coptidis*) and Huang Bai (*Cortex phellodendri*) and products containing the alkaloid Berberine has been prohibited in Singapore since 1972 based on sporadic cases and circumstantial evidence that Berberine could cause kernicterus in glucose-6-phosphate dehydrogenase (G6PD) deficient neonates. This ban was lifted four decades later, after an extensive review process by the local authority. Evidence came from scientific publications and surveillance of adverse reactions reported in the Asian countries which practise TCM; all of which did not suggest major safety concerns when Berberine was used appropriately, with the provision that it should still be avoided in infants and G6PD deficient individuals of all ages as well as pregnant and breastfeeding women [47, 48]. This illustrates that continuous research and review of herb toxicity against its therapeutic potential based on current evidence are important in guiding the rational use of herbs.

### 6.2. Herb-Drug Interactions

In communities where Western medicine is the primary mode of treatment and TCM is supplementary, there is a high prevalence for concomitant use of both drugs and herbs, raising the safety concern of potential herb-drug interactions. Interactions between drugs and herbs can generally occur at the pharmacodynamics level, whether the herbs may potentiate or antagonize the biological activities of the drugs by acting on the same target or at the pharmacokinetics level, where herbs may affect the absorption, distribution, metabolism, or excretion of the drugs [49, 50]. Specifically, herbs can induce or inhibit cytochrome P450 family proteins which are the main metabolizing enzymes or interfere with the efflux activity of P-glycoprotein which can actively pump absorbed drugs back into the intestinal lumen, thus affecting the bioavailability of concomitant drugs. This is of particular concern in patients who are taking drugs with narrow therapeutic index, for example, warfarin, cyclosporine A, and phenytoin, where certain herbs may potentially result in undesirable consequences of toxic effects or therapeutic failure [49, 50]. While such interactions are better studied in herbs used in Western folk medicine, for example, St John's Wort, Echinacea, and Milk thistle [49, 51], clinical evidence for the interaction potential of TCM herbs with drugs is hugely lacking and needs to be established clearly.

An example of possible adverse interaction is that between Danshen (*Salviae miltiorrhizae*) and warfarin, as demonstrated by some published evidences. Danshen is a TCM herb with “Blood-invigorating” property commonly used in cardiovascular conditions. There are case reports of patients stable on warfarin experiencing elevated international normalized ratio and bleeding after consuming Danshen-containing formulations [52, 53]. Preclinical studies investigating the interaction between Danshen and warfarin reported that the herb extract inhibited warfarin hydroxylation and increased its concentration in rats [54]. Furthermore, there were* in vitro* studies reporting that Danshen extract inhibited platelet aggregation [55]. Such evidences serve to provide preliminary but crucial information alerting on the potential problems with certain herb-drug pairs, which will mandate close monitoring in both clinical trials and real-life practice involving such combinations.

Contrary to the common assumption, not all herb-drug interactions are necessarily harmful. For instance, a study investigating the potential pharmacokinetic and pharmacodynamics interactions of a TCM formula CMF1 (Yinqiaosan and Sangjuyin) with Oseltamivir found that the antiviral activity of the Oseltamivir was enhanced by the herbal combination in healthy volunteers (*n* = 14) [56]. This suggests that the combined use of CMF1 and Oseltamivir could achieve a better therapeutic effect against influenza without compromising on safety. That said, more studies need to be done in this area to establish the information needed to guide the appropriate use of herbs in patients on concomitant drugs so that the therapeutic effect of either is not compromised and undesired toxic side effects can be avoided.

## 7. Approaches of Some Asian Countries

There are plenty of* in vitro* and animal studies exploring herb-drug interactions. However, much variation exists among the studies in aspects such as the methodology used and dose of herb tested resulting in inconclusive interpretations of the results collectively. Interpretation of these findings is further complicated by the incongruence of results, where the same herb could be reported in different studies to have opposing effects. Moreover, it is difficult to assess the clinical significance of these preliminary research findings. Nonetheless, it is essential that we maximize the use of what is available such that this mass of evidence is systematically collected and transcribed into meaningful databases to provide clinicians with an objective assessment of the current evidences and assist them in making an informed decision. With this as the main objective, we in the Singapore General Hospital established a Traditional Medicine Information Service (TMIS) in 2011 as an extension to the Drug Information Service in the Department of Pharmacy, with the committee formed by a medical doctor, pharmacists, and licensed TCM practitioner. TMIS provides evidence-based information on traditional medicines (TCM, Jamu, Ayurveda herbal medicine, etc.) by conducting literature searches and critically reviewing the current evidences. It has embarked on the development of a warfarin-herb interaction database which serves as readily available information for reference by pharmacists within the hospital. At the National University of Singapore, an online oncology drug interaction database “OncoRx” [57] has been developed by an informatics team led by a pharmacist to allow quick searches on interactions between different combinations of anticancer agents and herbal medicines.

In addressing the safety issue of TCM herbs, in Hong Kong, a multidisciplinary team consisting of a pharmacist, a chemical pathologist, a scientific officer, and a physician was formed to provide an advisory service on herbal safety to healthcare professionals of all public hospitals since 2000 [42]. As part of the investigation of adverse events associated with herbal products, the products and biologic samples would be screened for the presence of synthetic drugs and natural toxins, for example, aconitine, ephedrine, and aristolochic acids using various analytical tools [42].

On the national level, various Asian countries have their own adverse reaction reporting and capturing systems for TCM, mostly based on voluntary reporting. The Chinese SFDA has its system established in 1989 [58] and Taiwan has a similar reporting system established in 1998 [59], relatively late considering the long history of use of TCM in both countries. In Hong Kong, this aspect is governed by the territory-wide Drug and Poisons Information Bureau [42], while in Singapore the Health Sciences Authority has an Adverse Event Monitoring unit for the reporting and investigation of dubious products suspected to be linked with the adverse events [60]. These pharmacovigilance efforts have the primary objectives of ensuring public safety, but their databases built up over time should also be an invaluable source of preliminary evidence and serve as the basis for further laboratory and clinical research to establish definitive evidence of adverse reaction and herb-drug interactions.

## 8. Future Directions

Many issues surrounding the efficacy and safety of TCM use in modern society remain unresolved and these hinder the potential benefits of TCM from being safely and effectively harnessed. Applying EBM to TCM is vital for the traditional practice to gain acceptance by Western medical practitioners and become an integral component of the healthcare system. This is a necessary step when exploring TCM as cost-effective treatment options to help tackle healthcare issues such as functional diseases, chronic illnesses in ageing populations, and rising healthcare costs. High quality evidences via RCTs assessing efficacy and safety are needed to substantiate the use of TCM in suitable conditions.

However, EBM should not be rigidly imposed onto TCM but rather adapted with flexibility taking into consideration the unique characteristics of TCM practice. As advocated in a review by Tang, a pragmatic approach towards TCM research by adopting an efficacy-driven approach instead of the conventional mechanism-based approach may be more applicable to TCM. This approach starts with evaluating safety and efficacy in humans by RCTs, from which efficacious interventions are identified before undertaking studies on mechanisms and active substances. This would avoid unnecessary basic research on inefficacious interventions and better channel resources to further study beneficial treatments. Most importantly, this strategy may provide high-level evidence for the expedited recognition of effective TCM interventions [61].

With the advancement of technology and research tools, continuous effort in conducting rigorous research into toxicities and drug-interacting potential of herbs as well as correlation of laboratory findings with clinical observations are essential. This provides new insights about the herbs to help us utilize the herbs in a more rational and safe way in the modern clinical setting. Stringent quality control in the manufacturing of herbal products, strict regulatory controls on TCM herbal products, and active safety surveillance to detect adverse events are crucial to safeguard the risks that TCM users are exposed to.

There are many challenges in applying EBM to TCM, but these can be overcome by various approaches. In the era of advanced technology and EBM, with the wise application of basic and clinical research methodology to provide the relevant evidence, TCM as an ancient science and art is poised to be brought to a greater height for its ultimate mission of benefiting human health.
